# Multiple cardiac rhabdomyomas not associated with tuberous sclerosis in a dizygotic twins: a case report

**DOI:** 10.1186/s13256-021-02943-x

**Published:** 2021-07-05

**Authors:** Koji Yamamoto, Yohei Maki, Yuichiro Sato, Hiroyuki Tanaka, Tsuyoshi Fukushima, Junko Ushijima, Seishi Furukawa, Hiroshi Sameshima, Hiroaki Kataoka

**Affiliations:** 1 Section of Oncopathology and Regenerative Biology, Department of Pathology, Faculty of Medicine, University of Miyazaki, 5200 Kihara, Kiyotake, Miyazaki, Japan; 2 Department of Obstetrics and Gynecology, Faculty of Medicine, University of Miyazaki, Miyazaki, Japan; 3 Department of Diagnostic Pathology, Miyazaki University Hospital, University of Miyazaki, Miyazaki, Japan

**Keywords:** Cardiac rhabdomyoma, Dizygotic twin, Tuberous sclerosis complex, Autopsy

## Abstract

**Background:**

Rhabdomyomas comprise the majority of cardiac tumors in fetuses and are found in association with tuberous sclerosis complex. More than 90% of fetuses and neonates with multiple cardiac rhabdomyomas have signs of tuberous sclerosis complex. However, solitary cardiac rhabdomyoma cases are largely unrelated to tuberous sclerosis complex. Here, we report a case involving multiple cardiac rhabdomyomas not associated with tuberous sclerosis complex in a dizygotic twin.

**Case presentation:**

A 36-year-old Japanese woman was diagnosed with a dizygotic twin pregnancy in the first trimester. Consistent with dizygosity, the fetal sex was discordant (male and female). At 27 weeks of gestation, hydrops and multiple echogenic cardiac masses were noted in the male baby, with the largest mass measuring 34 × 30 mm. The female fetus appeared normal. The cardiac masses enlarged gradually with the progression of the hydrops. At 32 weeks of gestation, intrauterine death of the male fetus was confirmed. The next day, autopsy of the male fetus was performed after cesarean section. Three well-demarcated white-tan-colored nodules were formed in the ventricular walls and interventricular septum, with the largest nodule (40 × 30 mm) in the left ventricular wall. Histologically, these lesions were diagnosed as rhabdomyomas.

**Conclusions:**

We encountered a case involving multiple cardiac rhabdomyomas arising in one of dizygotic twin fetuses. Unlike most reported cases of multiple cardiac rhabdomyomas, this case was not accompanied by tuberous sclerosis complex. To the best of our knowledge, this is the first case report of multiple cardiac rhabdomyomas that developed in only one of dizygotic twins in the English literature.

## Background

Rhabdomyoma is the most common fetal cardiac tumor, accounting for 50–80% of all primary pediatric tumors [1, 2]. Approximately 90% of intracardiac tumors found *in utero* or during the neonatal period are rhabdomyomas [3]. Fetal cardiac rhabdomyoma usually increases in size until birth and may be accompanied by fetal hydrops, intrauterine fetal death, and sudden infant death. However, it gradually regresses after birth in approximately half of the cases [4]. The decrease in tumor size may be caused by decreased maternal estrogen levels [5]. These tumors grow to their largest size during the fetal period, shrink with age, and may even disappear completely [6]. Therefore, patients are usually followed without surgery. However, surgical resection may be required to prevent sudden death in patients with serious symptoms, such as severe ventricular outflow tract obstruction or arrhythmias [7]. Cardiac rhabdomyomas can be solitary or multiple. Cardiac rhabdomyomas commonly present with tuberous sclerosis complex (TSC), and more than 90% of multiple cardiac rhabdomyomas are associated with TSC [8, 9]. In contrast, the risk for the development of TSC with solitary cardiac rhabdomyomas is lower than that with multiple cardiac rhabdomyomas [9].

This report documents an autopsy case involving multiple cardiac rhabdomyomas in a dizygotic twin fetus lacking any signs or family history of TSC.

## Case presentation

**Fig. 1:**
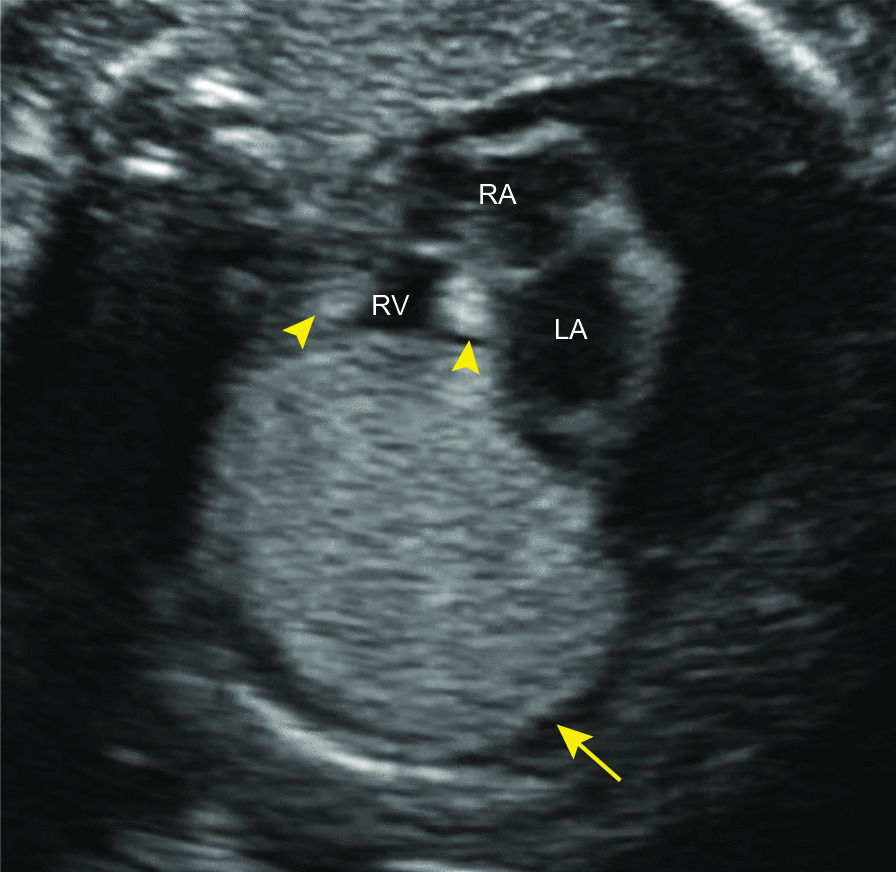
Fetal echocardiography at 26 weeks. A large left ventricular apical mass (arrow) and small nodules in the interventricular septum and right ventricular wall (arrowheads) were observed. *RA* right atrium, *RV* right ventricle, *LA* left atrium

**Fig. 2:**
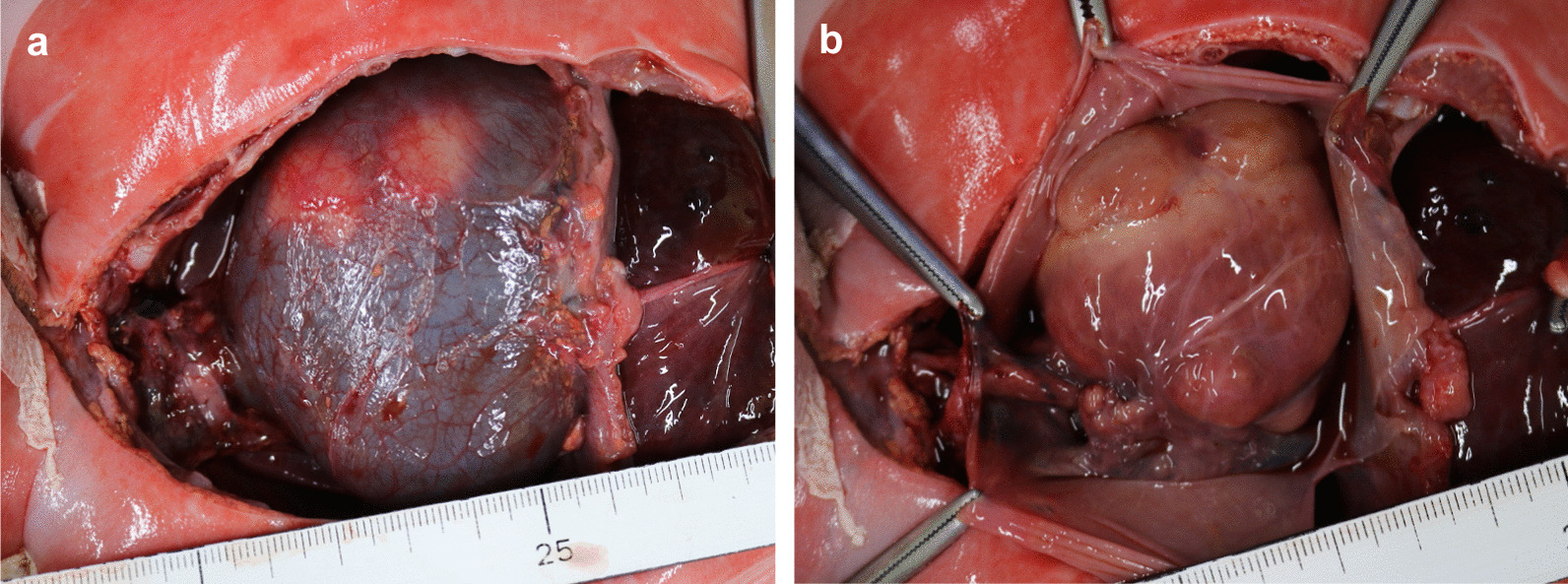
Macroscopic findings at autopsy. **a** The pericardial cavity was prominently expanded, and whitish cardiac nodules of a left ventricular lobulated mass are observed from the surface. **b** Macroscopic findings after pericardial incision. An enlarged heart (size: 4 × 3 × 2.5 cm) with multiple tumor nodules is seen

**Fig. 3:**
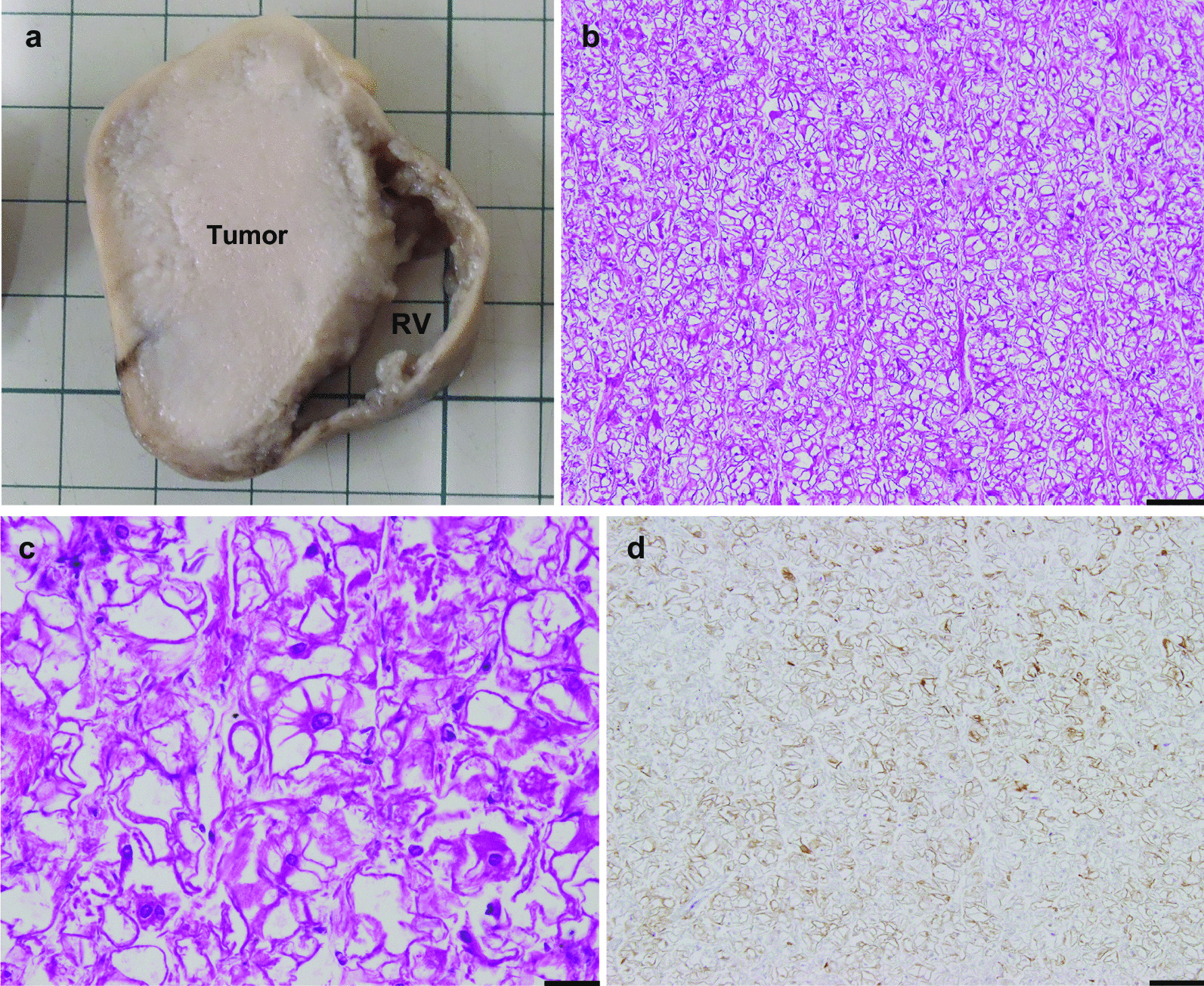
Histology of the cardiac tumor. **a** The cut surface of the largest tumor after formalin fixation. Tumor filling the left ventricular cavity. **b** The tumor was composed of the vacuolated tumor cells (HE, ×40). Bar, 200 μm. **c** Higher magnification of the tumor tissue, showing the presence of so-called spider cells (HE, ×400). Bar, 20 μm. **d** Immunohistochemical staining for desmin. The tumor cells were strongly positive for desmin. Bar, 200 μm. *RV* right ventricle, *HE* hematoxylin and eosin

A 36-year-old Japanese woman, gravida 1 para 0, was diagnosed with dichorionic diamniotic twin pregnancy in the first trimester. On ultrasound examination, each fetus was surrounded by a thin amnion, which was divided by a thick membrane (chorion). The sexes were discordant (male and female), consistent with dizygosity. The patient was a housewife and did not drink alcohol and smoke. The patient was healthy and had no history of diabetes or hypertension. At 27 weeks of gestation, she was referred to the Department of Obstetrics and Gynecology at Miyazaki University Hospital because fetal ultrasonographic examination revealed hydrops and multiple echogenic cardiac masses in the male fetus (Fig. 1). The other organs, including the intracranial tissues, appeared to be normal. No obvious abnormalities were observed in the female fetus. The cardiac masses of the male fetus gradually enlarged, and the fetal hydrops worsened. At 32 weeks of gestation, the physician confirmed intrauterine death of the male fetus. She delivered a stillborn male baby and a normal-appearing female baby by cesarean section the next day. The female baby weighed 1535 g, and her Apgar scores were 7 and 8 at 1 and 5 minutes, respectively. Postnatal echocardiography of the female baby did not reveal any cardiac abnormalities. Neither congestion, hemorrhage, nor infection was observed in the placenta. An autopsy was then performed on the male baby. His body weight was 2320 g, with no apparent external malformations. The pericardium had expanded prominently, pushing both lungs away to the dorsal side (Fig. 2a). The pericardial cavity contained 40 mL of effusion, and the inner surface was smooth without adhesions. The largest cardiac tumor was observed in the left ventricular wall, forming a 40 × 30 mm multilobulated mass that compressed the left ventricular cavity significantly (Fig. 2b). Other tumor nodules, measuring 7 × 5 mm and 8 × 3 mm, were also present in the right ventricular wall and interventricular septum, respectively. The cut surface of the nodule was white, and the tumor occupied the left ventricular cavity (Fig. 3a). Histologically, these masses were composed of a proliferation of polygonal tumor cells with small centrally placed nuclei and abundant clear cytoplasm (Fig. 3b). The vacuolated “spider cells,” which possessed strands of cytoplasm emanating from the nucleus, were intermingled (Fig. 3c). Tumor cells were immunohistochemically positive for anti-desmin monoclonal antibody (Fig. 3d). The tumor cells lacked mitotic activity, and positive cells were hardly detectable by immunohistochemical staining for the Ki-67 monoclonal antibody. Necrotic changes were not observed. The pathological diagnosis was cardiac rhabdomyoma. The cause of fetal death was thought to be circulatory failure due to multiple cardiac rhabdomyomas, particularly caused by the largest tumor in the left ventricular wall. No evidence of TSC was observed in either parent. His twin sister did not show any signs of TSC after birth. The final diagnosis was multiple cardiac rhabdomyomas that occurred in the absence of TSC.

## Discussion and conclusions

We showed multiple cardiac rhabdomyomas in one of the dizygotic twins. Most cases with multiple cardiac rhabdomyoma have been reported to be associated with TSC. Compared with previous literature, this report presents a very interesting case of multiple cardiac rhabdomyoma with no signs of TSC in one of the twins.

Fetal cardiac tumors are rare, occurring in 0.14% of pregnancies, and rhabdomyoma is the most common fetal cardiac tumor [10]. This tumor is congenital, and patients are commonly diagnosed prenatally in infancy, although a few of these tumors were first diagnosed in older children [7]. Other fetal cardiac tumors include fibromas, hemangiomas, and teratomas [9]. The most common locations for cardiac rhabdomyoma are the left ventricle and interventricular septal areas, and the incidence of cardiac rhabdomyoma in the atria is low [11]. In our case, multiple rhabdomyomas were located in both the ventricular walls and interventricular septum. Macroscopically, the tumors were well defined, noncapsulated, and white-tan in color. The largest tumor was located in the left ventricle, with a maximum diameter of approximately 40 mm. The macroscopic appearance of the largest tumor was a multinodular form in which several nodules appeared to be fused; however, it appeared as a single nodule on the cut surface. In the literature, one report described a case involving a giant cardiac rhabdomyoma measuring > 5 cm in a newborn infant [12].

The symptomatic presentation of cardiac rhabdomyoma is variable, including cyanosis, hydrops, heart failure, and arrhythmias, depending on the number and location of the tumors [6]. Previous reports revealed that obstruction of the intracardiac flow, alteration of the atrioventricular valve function with consequent regurgitation, arrhythmia, cardiac dysfunction, and hydrops are indicators of poor prognosis [13]. Moreover, a tumor size > 20 mm was significantly associated with the risk of adverse outcomes [14]. We presume that the current case had a dismal prognosis due to the large size of the left ventricular tumor and the subsequent disturbance of intracardiac blood flow and hydrops.

One of the most important consequences of cardiac rhabdomyoma is its association with TSC, a genetic disorder with autosomal dominant inheritance. This disease is characterized by widespread hamartomas in several organs, including the heart, skin, brain, kidneys, pancreas, and lungs. Retrospective reviews or case reports indicate that approximately 95% of cases involving multiple cardiac rhabdomyomas are accompanied by TSC [8]. However, in the present case, the parents had no family history of TSC, and his twin sister had no signs of TSC. Moreover, the mother and father were not relatives.

To date, only five cases of cardiac rhabdomyoma have been reported in twins [15–19] (Table 1). One study reported that one of the twins had a single cardiac rhabdomyoma, and that case was not accompanied by TSC [16]. On the other hand, all of the other four reports showed multiple cardiac rhabdomyomas accompanied by TSC in both twins [15, 17–19]. In the current case, we identified multiple cardiac rhabdomyomas in one of the dizygotic twins. To the best of our knowledge, this is the first case report of multiple cardiac rhabdomyomas that developed in a twin without any signs of TSC.

**Table 1:** Previous reports of twin cardiac rhabdomyomas, including this case

	Case	Sex	Twin	Rhabdomyoma	TSC	Outcome
Gushiken *et al*. [15]	Twin A	Unknown	Monozygotic	Multiple	+	Alive
	Twin B			Multiple		Alive
Verma *et al*. [16]	Twin A	Male	Dizygotic	Single	−	Death
	Twin B	Female		–		Alive
Chadha *et al*. [17]	Twin A	Unknown	Dizygotic	Multiple	+	Death
	Twin B			Multiple		Alive
Colosi *et al*. [18]	Twin A	Male	Dizygotic	Multiple	+	Alive
	Twin B	Female		Multiple		Alive
Ben Salem *et al*. [19]	Twin A	Unknown	Unknown	Multiple	+	Alive
	Twin B			Multiple		Alive
Our case	Twin A	Male	Dizygotic	Multiple	−	Death
	Twin B	Female		–		Alive

## Data Availability

The datasets used and analyzed during the current study are available from the corresponding author upon reasonable request.

## Abbreviations

TSC: Tuberous sclerosis complex
TSC: Tuberous sclerosis complex
